# Acute Myocardial Infarction Due to Spontaneous Coronary Artery Dissection and Plaque Rupture

**DOI:** 10.7759/cureus.8063

**Published:** 2020-05-11

**Authors:** Sana Riaz, Mostafa Vasigh, Emad Mogadam, Devjani Ganesan, Debanik Chaudhuri

**Affiliations:** 1 Internal Medicine, State University of New York (SUNY) Upstate Medical University, Syracuse, USA; 2 Cardiology, State University of New York (SUNY) Upstate Medical University, Syracuse, USA; 3 Cardiology, University of Southern California, Los Angeles, USA; 4 Interventional Cardiology, State University of New York (SUNY) Upstate Medical University, Syracuse, USA

**Keywords:** chest pain, acute st-elevation myocardial infarction, spontaneous coronary artery dissection, mortality

## Abstract

Myocardial infarction (MI) can be secondary to atherosclerotic coronary artery disease (ACAD) and non-atherosclerotic coronary artery disease (NACAD). The common cause of NACAD in young females is spontaneous coronary artery dissection (SCAD). We present a case of SCAD and plaque rupture leading to MI.

## Introduction

Spontaneous coronary artery dissection (SCAD) is a rare cause of acute coronary syndrome (ACS), with an angiographic incidence of 0.1%-1.1% and accounts for up to 35% of myocardial infarctions (MI) in women ≤ age 50 [1,2]. We present a case of a 45-year-old female with no prior cardiac history admitted with chest pain and noted to have the rare co-existence of SCAD and atherosclerotic coronary artery disease (ACAD). Our case also highlights the importance of early recognition of ACS and prompt intervention leading to improved outcomes as noted in our patient.

## Case presentation

**Figure 1:**
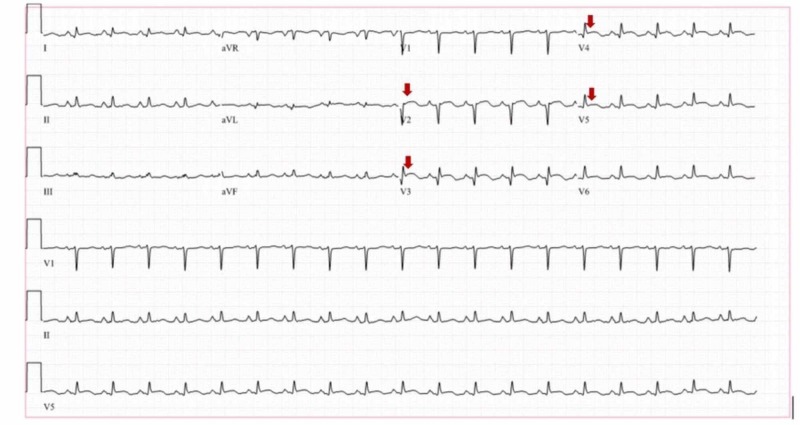
Electrocardiogram demonstrating ST-segment elevations in the anterior leads (arrows)

A 45-year-old female, active smoker with no prior cardiac disease presented with acute onset chest pain. She was in acute distress. The chest pain was central with radiation to the left arm and was associated with shortness of breath. She had no family history of MI at age less than 50 years and no sudden cardiac death. Vitals signs on admission: blood pressure 90/80 mmHg, heart rate 100 beats/minute, and respiratory rate 20 breaths/minute. The patient was alert but lethargic. Heart sounds were normal, and no murmurs or added sounds were appreciated. Auscultation of the lung revealed bilateral air entry on initial evaluation. Laboratory work on admission showed normal renal function, normal complete blood count, except for leukocytosis of 13.6 x 10^3^/µL. She had elevated troponin T of 1.74 ng/mL and brain natriuretic peptide (BNP) of 8158 pg/mL. Urine toxicology was negative. The electrocardiogram showed ST elevations in the anterior leads (Figure 1).

**Figure 2:**
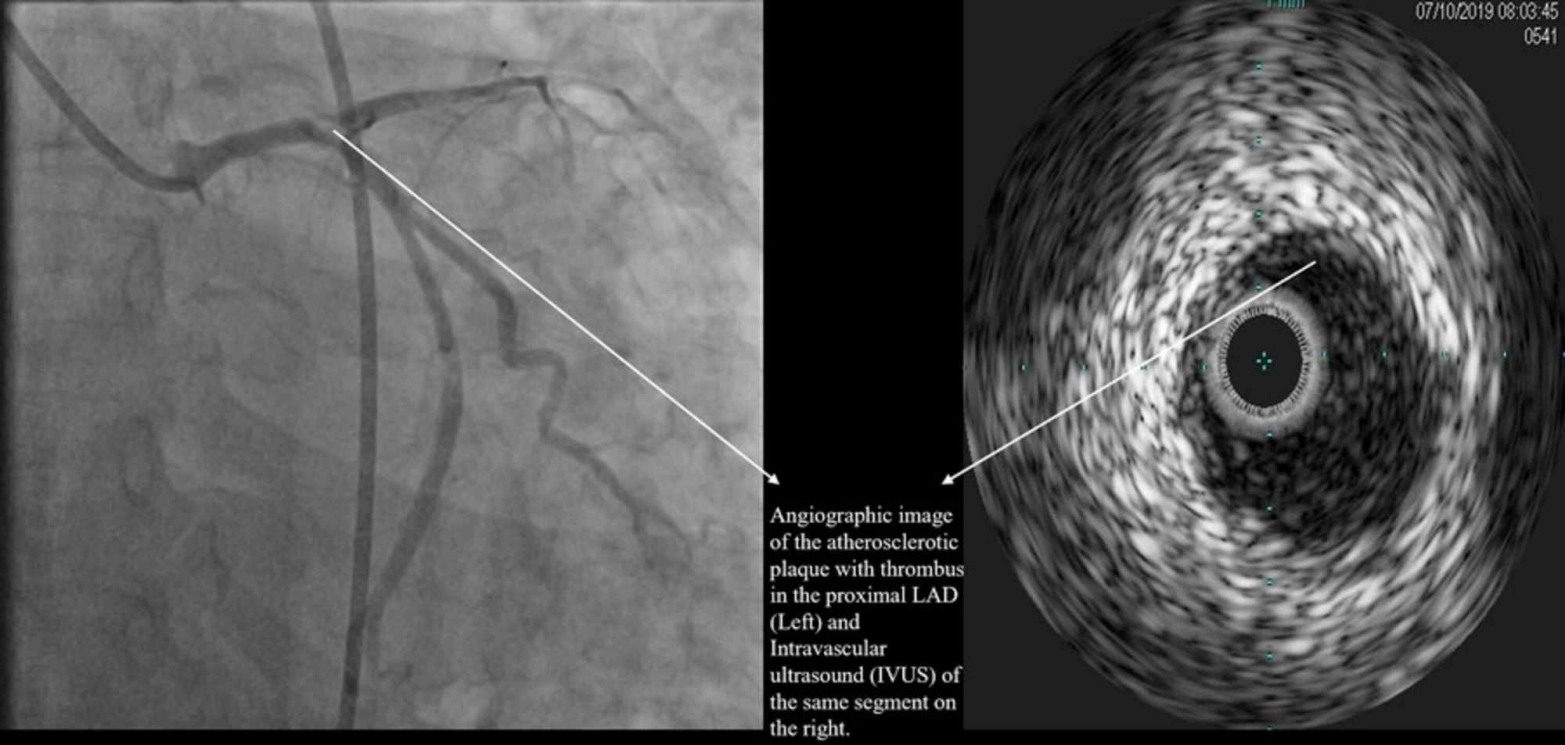
Proximal left anterior descending artery with atherosclerotic plaque and thrombus LAD: Left anterior descending.

She was taken for emergent cardiac catheterization, which showed angiographically normal right coronary artery, a ruptured plaque with thrombus, and 90% stenosis involving the ostial left anterior descending artery (LAD) (Figure 2).

**Figure 3:**
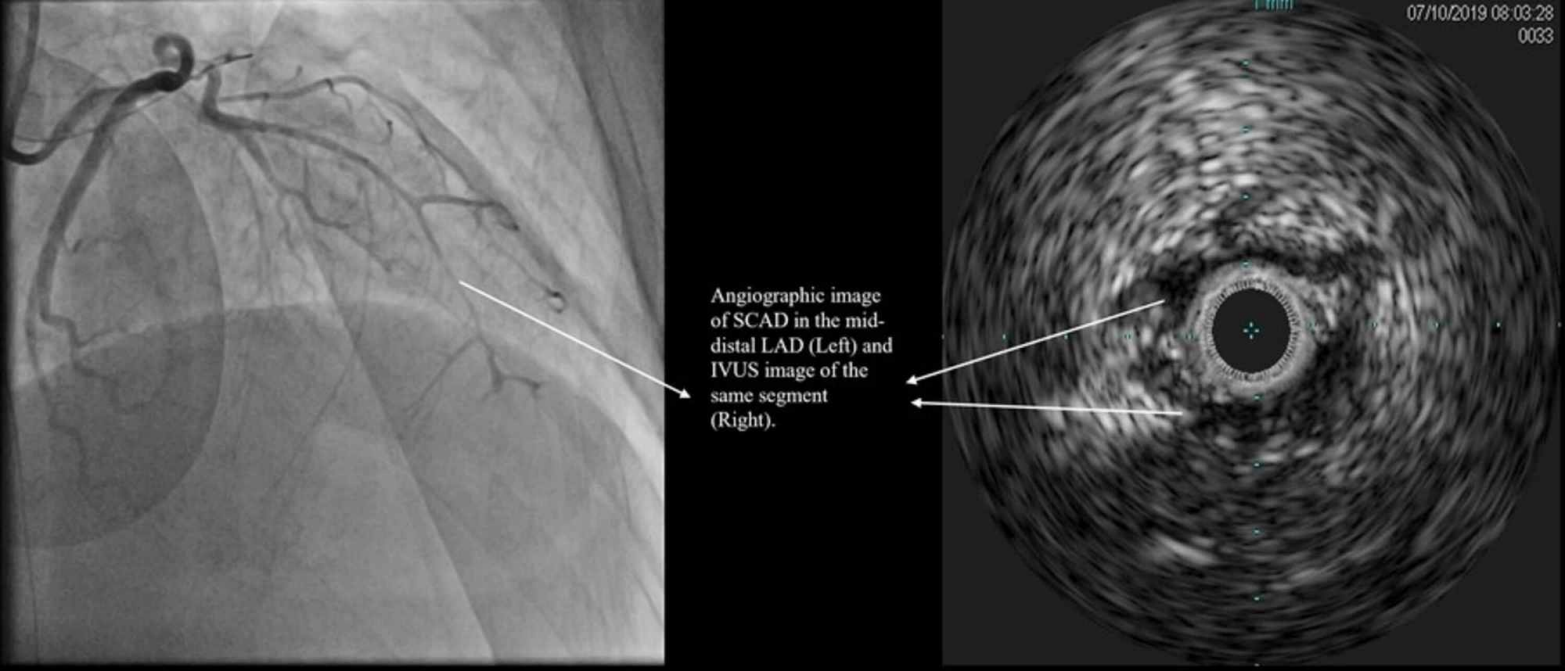
Spontaneous coronary artery dissection of the mid-distal left anterior descending artery SCAD: Spontaneous coronary artery dissection; LAD: Left anterior descending; IVUS: Intravascular ultrasound.

The mid-LAD showed diffuse narrowing, and apical LAD was completely occluded. The angiographic appearance of the lesions in mid-distal LAD was consistent with SCAD (Figure 3).

This was subsequently confirmed on intravascular ultrasound (IVUS). The patient was in refractory cardiogenic shock (presence of low cardiac index and started on norepinephrine, vasopressin, and epinephrine) and required mechanical circulatory support (Impella, Aachen, Germany). A drug-eluting stent was placed in the proximal LAD. The apical occluded LAD was not crossed with a wire. The mid-LAD was treated with prolonged low-pressure balloon inflation.

Echocardiogram post- catheterization revealed akinetic anterior wall and an ejection fraction of 20%-25%. She was gradually weaned off the vasopressors, and the Impella was removed 48 hours post-cardiac catheterization. She was medically optimized, and after hemodynamic stabilization, she was discharged home on dual antiplatelet therapy, high-intensity statin, lisinopril, and metoprolol succinate. Spironolactone was not initiated during the hospital stay due to low blood pressure.

The patient was followed up as an outpatient in two weeks and underwent a stress echocardiogram. Her left ventricular ejection fraction (LVEF) improved to 35%-40%, and she achieved 5.6 metabolic equivalents (METS). She was subsequently referred for cardiac rehabilitation and continues to follow with cardiology. 

## Discussion

Cardiovascular disease is the leading cause of death in women in the United States. The mortality due to MI in women younger than 50 years of age is two-fold higher than age-matched men. In previous studies, including a study by Saw et al., ACAD has been noted to be the most common etiology leading to MI [3]. However, non-atherosclerotic coronary artery disease (NACAD) is also an important cause for MI, and SCAD attributes to the majority of the NACAD cases [3]. SCAD is the dissection of the epicardial coronary artery that is not associated with atherosclerosis and trauma. Patients present with acute coronary syndrome (ACS) from coronary artery obstruction due to intramural hematoma rather than atherosclerotic plaque rupture or intraluminal thrombus. The LAD is the most affected artery (32%-46% of cases). Medical management of SCAD is similar to ACAD, however, the decision regarding percutaneous coronary intervention depends on multiple factors including perceived risk of complete coronary artery dissection and total occlusion and/or presence of hemodynamic compromise and cardiogenic shock [2,4]. It has been noted, based on a review by Saw et al., that the long term major adverse cardiac events (MACE) rates in patients with SCAD are 15% to 37% at five to seven years and almost 50% at 10 years [5]. Early recognition of SCAD is imminent since it is associated with a high mortality rate of about 50% [1].

Our case emphasizes the importance of a thorough evaluation of young women with no prior cardiac history and no cardiovascular risk factors presenting with acute onset chest pain. Since it has been observed that young women (less than 55 years of age) presenting with ACS, were most likely to be discharged from the emergency room with a missed diagnosis than other age groups or sex [3]. Our patient presented for the first time with chest pain, and on evaluation, she was found to have both SCAD and plaque rupture. However, appropriate and timely intervention can significantly decrease morbidity and mortality, as seen in our case [2]. The patient was stabilized and discharged home four days after her admission. 

Additionally, our case is also unique since there are no case reports or literature reported with the coexistence of both SCAD and plaque rupture simultaneously leading to MI, based on our database search through PubMed and Google Scholar.

## Conclusions

NACAD and ACAD are associated with risk factors; however, women under the age of 55 years without known risk factors for either entity should be evaluated when they present with acute onset chest pain. Additionally, risk stratification scores such as the history, ECG, age, risk factors, and troponin (HEART) score can facilitate decision making as it is also useful in patients with low risk of ACS.
